# The influence of the maternal peer group (partner, friends, mothers’ group, family) on mothers’ attitudes to obesity-related behaviours of their children

**DOI:** 10.1186/s12887-019-1726-x

**Published:** 2019-10-16

**Authors:** Adrian J. Cameron, Emma Charlton, Adam Walsh, Kylie Hesketh, Karen Campbell

**Affiliations:** 1 Global Obesity Centre, Institute for Health Transformation, Deakin University, Geelong, Australia; 2 Institute for Physical Activity and Nutrition, Deakin University, Geelong, Australia

**Keywords:** Peers, Infant, Feeding, Physical activity, Sedentary behaviour, Early childhood

## Abstract

**Background:**

Relationships with others can have an impact on the attitudes of new mums to the obesity-related behaviours of their children. The aim of this study was to understand the degree to which other new mums (from their mothers’ group), friends, partners, and other family members have an influence on maternal attitudes to child feeding, physical activity and television viewing behaviours in order to more accurately target obesity prevention interventions.

**Methods:**

In a retrospective cohort study design using data from the InFANT randomized controlled trial, first-time mothers (*n* = 307) from Melbourne, Australia were asked in 2012–13 how much of an influence their partner, friends, mothers’ group and family were on their attitudes to their pre-school aged child’s feeding, physical activity and television viewing behaviours. The level of influence was examined using chi-square tests, t-tests, and analysis of variance, stratified by maternal education, age and body weight. We also examined associations between the influence of others on maternal attitudes and actual behaviours including breastfeeding duration, age at introduction of solid food and time their child spent outside.

**Results:**

Mothers rated partners as having the strongest influence on their attitudes toward all obesity-related behaviours. The percentage reporting partners as a major influence were 28.7% (95% CI 23.8,34.0), 33.1% (28.0, 38.6) and 24.2% (19.6, 29.3) for child feeding, physical activity and television viewing, respectively. More highly educated mothers rated social connections as more influential than less educated mothers. The influence of partners on attitudes toward child feeding was associated with longer breastfeeding duration.

**Conclusions:**

Mothers rated partners as a powerful influence on their attitudes toward the obesity-related behaviours of their pre-school children, suggesting that partners could be an important target of obesity-prevention initiatives. Since less educated mothers reported peers and family as a much weaker influence on their attitudes to obesity-related behaviours than more educated mothers, equity should be taken into consideration when contemplating obesity-prevention interventions that target mothers’ groups.

## Background

Childhood overweight and obesity are highly prevalent globally, with one in four Australian children overweight or obese [1], and a higher prevalence in socioeconomically disadvantaged communities [2]. Overweight and obesity have consequences for physical, cognitive and motor development in young children [3] and are linked to psychological co-morbidities, later life obesity and numerous chronic diseases [4].

Childhood is a critical period in which eating and physical activity behaviours are developed and there is evidence that these modifiable behaviours track across childhood and adolescence [4]. Parents are a crucial influence on these behaviours of their young children with knowledge about healthy diets [5], modelling healthy eating [6] and feeding practices such as sharing family meals [7] all positively influencing child diet quality. Physical activity engagement in children is also heavily influenced by parents’ behaviours [8] with a positive association apparent between the screen-time use of parents and their children [9].

Parenting practices of mothers are shaped by a variety of influences including peers and the wider social environment [5]. Fathers are a particularly strong influence and are increasingly responsible for the care of their children. Emerging evidence supports a correlation between father and offspring diets [10] and activity [11], with paternal feeding strategies [12] and food-related knowledge also linked to child diet quality [13].

Support of other close family members, particularly grandmothers, has been shown to influence both breastfeeding and child diet quality, although the literature is not conclusive [14], and some studies suggest that grandmothers may have a greater influence in societies with stronger generational links [15].

Outside of the family, mothers’ groups provide an important source of support for first-time mothers and these ‘true peers’ (i.e. other mothers of similar-aged children) have been shown to influence both breastfeeding [16] and physical activity behaviours [17]. Other friends can also influence behaviour, [18] although mothers have reported that they mainly use their friends to determine what to avoid doing when it comes to feeding their child [19].

Using social network analysis, Christakis and Fowler found that obesity among peers increases a person’s chance of becoming obese [20]. The clustering of weight within social networks [21] and similarities in eating patterns between family members and friends [22] provide further evidence that obesity-related behaviours are socially influenced. Social network interventions to reduce obesity may also be more effective than targeting individuals alone [23].

The aim of this study was to investigate the relative influence of first-time mothers’ partners, other family, friends and mothers’ group on their attitudes to infant feeding, physical activity, and TV viewing behaviours. Whether associations are influenced by maternal age, education, BMI and mothers’ group attendance, and whether the influence of others is associated with actual child behaviours are also explored.

### Methods

This study uses a retrospective cohort study design, with data from a cohort of first-time parents involved in the Melbourne Infant Feeding, Activity and Nutrition Trial (InFANT) Program, a cluster-randomised controlled trial testing an intervention (six two-hour sessions delivered by a dietitian) that aimed to improve children’s diets, promote active play and reduce television viewing. Data for the current study was obtained from parent questionnaires administered at a two-year follow-up of this trial cohort (including parents from both treatment and control groups) when the children were aged 3.5 years [24].

### Study design and sample

The InFANT intervention protocol has been previously reported [25] but in brief, the program was delivered quarterly over a 15-month period from June 2008–February 2010 to 542 parents (equal numbers in control and intervention groups) who were members of 62 randomly selected first-time parent groups in Melbourne, Australia. Children were aged approximately 4 months at baseline and 20 months at the end of the intervention. Parents were eligible for inclusion if they were consenting, first-time parents, who were members of a participating new parents group’ and able to communicate in English [25].

Participants (*n* = 307) were followed up in 2012–13, approximately two years after the intervention ceased to assess long-term effects [24]. Ethics approval was granted by the Deakin University Human Research Ethics Committee (2007–175). Written informed consent to participate was obtained from all participants.

### Data collection and measures

A questionnaire was mailed to participants who were asked to complete it prior to a home visit by a research assistant. Child height and weight were measured by research assistants at home visits. BMI z-score was calculated using the ‘zanthro’ add-on in Stata which uses WHO sex-specific growth standards [26]. Maternal height and weight measurements were also taken at home visits where possible or otherwise self-reported [24].

The results presented here relate to questions about external influences on child feeding, physical activity and TV viewing. These were purpose-designed questions (provided as Additional file 1) on which test-retest reliability and internal reliability analyses were performed. In relation to a) mothers’ group, b) friends, c) partner, and d) other family members (all potential influencers of behaviour), mothers were asked to rate on a four-point scale (where 1 = no influence, 4 = major influence) the following questions: 1) How much of an influence on your attitudes towards feeding your child have the following been? 2) How much of an influence on your attitudes towards your child’s television viewing have the following been? and 3) How much of an influence on your attitudes towards your child’s activity levels have the following been?

Maternal education was self-reported as: no formal education, year 10 or equivalent, year 12 or equivalent, trade/apprentice/certificate, certificate/diploma, University degree or higher degree.

For assessment of peer influence by education level, maternal age and maternal weight, dichotomous categories were created based on a) those with a University degree or higher, vs. those with lower levels of education, b) mothers less than 35 years of age vs. those over 35 years and c) healthy weight vs. overweight/obese (BMI ≥ 25 kg/m^2^) respectively.

To examine the association between peer influence and related behaviours, questions were asked about the following: 1) duration of breastfeeding, 2) age of introduction of solids, 3) time child spends outside on an average day over the last week, and 4) average number of serves of fruits (per day), vegetables (per day) and hot chips (per week, includes hot chips, French fries, wedges, fried potatoes) consumed by the child.

### Data analysis

Demographic statistics were calculated as mean (standard deviation or min/max), percentage or n as appropriate. The level of influence of each of mothers’ group, friends, partner and other family members was calculated (percentage reporting no influence, little influence, some influence or a major influence respectively, with 95% confidence intervals) for each of child feeding, child television viewing and child physical activity. Stratified analyses were conducted according to whether mother/child dyads were in the control or intervention arm of the InFANT program, whether they had attended a mothers’ group meeting in the last four weeks, and based on maternal education, age and BMI. Chi square tests were used to test for differences in the level of influence reported for each group. One-way analysis of variance (ANOVA) was used to test for associations between peer influence ratings and behaviours expressed as continuous variables (breastfeeding duration, time of introduction of solids, time spent outside) and t-tests were used to assess associations with behaviours expressed as categorical variables (consumption of various foods). Analyses of peer influence on behaviours were conducted for the whole sample, and then for the control group only to account for the potential impact of the InFANT intervention. Stata version 14.0 (StataCorp, College Station, TX, USA) was used for analysis.

## Results

Of 307 respondents, 306 were mothers with one father excluded. Demographic and body weight characteristics are presented in Table 1.

**Table 1:** Characteristics of Participants (*n* = 306) in the 2012–13 follow-up of the Melbourne Infant Feeding, Activity and Nutrition Trial (InFANT) Program

Marital Status (%)	
Married	81
Living in a de facto relationship	14.4
Separated	2.3
Divorced	0.3
Never married (single parent)	2.0
Age and BMI	
Child age (average, min, max)	3.61 (3.2–4.5)
Mother’s age (average, SD)	35.8 (4.3)
Child BMI z-score (average, SE)	0.62 (0.50)
Mother’s BMI (average, SD)	25.3 (5.6) ^a^
Education (%)	
No formal qualifications	0.5
Y10 or equivalent	4.4
Y12 or equivalent	13.8
Trade/apprenticeship/certificate	3.0
Certificate/diploma	18.6
University degree	35.5
Higher degree	24.1

^a^Pregnant women (*n* = 44) excluded from analysis of mother’s BMI

### Peer influences

The self-reported influence of different relationships on mothers’ attitudes toward each of child feeding, television viewing and physical activity behaviours is presented in Table 2. Partners were the strongest influence on attitudes toward each behaviour. Other family members (i.e. mothers’ parents, in-laws, aunties/uncles, cousins and siblings) were considered to be the next strongest influence, followed by friends. Overall, participants rated their mothers’ group as the weakest influence on their attitudes toward their child’s obesity-related behaviours. Of all the behaviours examined, attitudes toward child feeding were most likely to be influenced by mothers’ group peers, followed by child physical activity. Attitudes toward child television viewing was reported as being least likely to be influenced by any relationships.

**Table 2:** Self-reported influence of mothers’ group peers, friends, partner and other family on maternal attitudes toward child feeding, physical activity and television viewing behaviours. Data from the 2012–13 follow-up of the Melbourne Infant Feeding, Activity and Nutrition Trial (InFANT) Program

		No influence	A little influence	Some influence	Major influence	No partner^a^
Child feeding	Mothers group	41.3 (35.7, 46.9)	29.9 (24.9, 35.3)	24.8 (20.2, 30.0)	4.0 (2.2, 6.9)	
	Friends	27.6 (22.7, 32.9)	41.2 (35.7, 46.8)	28.2 (23.4, 33.6)	3.0 (1.5, 5.6)	
	Partner	10.3 (7.3, 14.3)	19.3 (15.2, 24.2)	40.0 (34.5, 45.6)	28.7 (23.8, 34.0)	1.7 (0.6, 3.9)
	Other family	20.0 (15.8, 24.9)	34.3 (29.1, 39.9)	39.3 (33.9, 45.0)	6.3 (4.0, 9.7)	
Child TV viewing	Mothers group	65.3 (59.7, 70.0)	21.3 (17.0, 26.4)	12.7 (9.3, 16.9)	0.7 (0.1, 2.6)	
	Friends	55.6 (49.9, 61.1)	28.8 (23.9, 34.2)	13.6 (10.1, 17.9)	2.0 (0.8, 4.3)	
	Partner	17.5 (13.6, 22.2)	25.2 (20.5, 30.3)	31.5 (26.4, 36.9)	24.2 (19.6, 29.3)	1.7 (0.6, 3.9)
	Other family	40.9 (35.4, 46.5)	33.9 (28.7, 39.4)	21.9 (17.5, 26.9)	3.3 (1.7, 6.0)	
Child activity	Mothers group	51.7 (45.9, 57.3)	26.3 (21.6, 31.6)	19.0 (14.9, 23.8)	3.0 (1.5, 5.6)	
	Friends	41.0 (35.5, 46.6)	33.0 (27.8, 38.5)	22.7 (18.2, 27.7)	3.3 (1.7, 6.1)	
	Partner	18.2 (14.2, 23.0)	16.6 (12.7, 21.2)	30.5 (25.5, 35.9)	33.1 (28.0, 38.6)	1.7 (0.6, 3.9)
	Other family	31.9 (26.8, 37.4)	30.9 (25.9, 36.0)	33.6 (28.4, 39.1)	3.7 (2.0, 6.4)	

^a^Note, percentages in the column “no partner” are the percentage of respondents who reported having no partner, meaning that this question was not applicable to them

### Moderating influences

Significant differences in who mothers rated as the strongest influence on attitudes toward obesity-related behaviours were found according to maternal education (Table 3). University educated mothers were more likely to report that their mothers’ group, friends and partners were an influence on their attitudes toward child feeding and child activity behaviours, than were non-University educated mothers (all *p* < 0.02). University educated mothers were also more likely to rate partners (*p* = 0.001) and other family (*p* = 0.032) as strong influences on their attitudes toward their child’s TV viewing compared to non-University educated mothers.

**Table 3:** Influence of mothers’ group peers, friends, partner and other family on maternal attitudes towards child feeding, physical activity and television viewing behaviours, by education level. Data from the 2012–13 follow-up of the Melbourne Infant Feeding, Activity and Nutrition Trial (InFANT) Program

			No influence	A little influence	Some influence	Major influence	No partner	p*
Child Feeding	Mothers’ Group	High school, trade/certificate/diploma, % (*n* = 118)	54.2	28.8	12.7	4.2	0.0	**< 0.0001**
		University (*n* = 180)	32.8	30.6	32.8	3.9	0.0	
	Friends	High school, trade/certificate/diploma, % (*n* = 121)	33.9	46.3	19.0	0.8	0.0	**0.004**
		University (n = 180)	23.3	37.8	34.4	4.4	0.0	
	Partner	High school, trade/certificate/diploma, % (*n* = 120)	16.4	25.0	34.5	24.1	3.4	**0.003**
		University (*n* = 180)	6.7	16.2	44.7	32.4	0.6	
	Other Family	High school, trade/certificate/diploma, % (*n* = 120)	25.0	35.0	34.2	5.8	0.0	0.27
		University (*n* = 180)	16.7	33.9	42.8	6.7	0.0	
Child Television Viewing	Mothers’ Group	High school, trade/certificate/diploma, % (*n* = 120)	73.3	18.3	8.3	0.0	0.0	0.069
		University (*n* = 180)	60.0	23.3	15.6	1.1	0.0	
	Friends	High school, trade/certificate/diploma, % (*n* = 122)	59.8	27.9	12.3	0.0	0.0	0.17
		University (*n* = 180)	52.8	29.4	14.4	3.3	0.0	
	Partner	High school, trade/certificate/diploma, % (*n* = 122)	27.1	28.0	23.7	21.2	3.4	**0.001**
		University (*n* = 180)	11.7	24.0	37.4	26.8	0.6	
	Other Family	High school, trade/certificate/diploma, % (*n* = 121)	49.6	26.4	22.3	1.7	0.0	**0.032**
		University (*n* = 180)	35.0	38.9	21.7	4.4	0.0	
Child Physical Activity	Mothers’ Group	High school, trade/certificate/diploma, % (*n* = 120)	62.5	21.7	14.2	1.7	0.0	**0.02**
		University (*n* = 180)	44.4	29.4	22.2	3.9	0.0	
	Friends	High school, trade/certificate/diploma, % (*n* = 120)	51.7	29.2	17.5	1.7	0.0	**0.015**
		University (*n* = 180)	33.9	35.6	26.1	4.4	0.0	
	Partner	High school, trade/certificate/diploma, % (*n* = 122)	29.7	16.1	28.0	26.3	3.4	**< 0.0001**
		University (*n* = 180)	11.2	17.3	33.0	38.5	0.6	
	Other Family	High school, trade/certificate/diploma, % (*n* = 121)	34.7	30.6	30.6	4.1	0.0	0.76
		University (*n* = 180)	30.0	31.1	35.6	3.3	0.0	

*p < 0.05 presented in bold

When comparing treatment and control groups, mothers who took part in the InFANT intervention were more likely to rate their mothers’ group as having influenced their attitudes toward their child’s feeding than their control group counterparts (*p* = 0.045). Of those in the intervention group, 34.4% rated their mothers’ group as some or a major influence on attitudes to feeding compared to 23.2% of controls. Mothers in the treatment and control groups were similar in their rating of the influence of partners, friends and other family on their attitudes toward all behaviours (all *p* > 0.05).

The self-reported influence of others on attitudes toward obesity-related behaviours according to recent attendance at a mothers’ group meeting is presented in Table 4. Those who had attended a meeting in the past four weeks rated their group to be a greater influence on child behaviours than those who had not attended during this period (*p* < 0.001 for child feeding, child TV viewing and child physical activity).

**Table 4:** Influence of mothers’ group peers on maternal attitudes to child feeding, physical activity and television viewing among mothers who attended a session < 4 weeks ago and those who attended > = 4 weeks ago. Data from the 2012–13 follow-up of the Melbourne Infant Feeding, Activity and Nutrition Trial (InFANT) Program

Behaviour	Last attended mothers’ group	No influence^a^	A little influence	Some influence	Major influence	p^†^
Child Feeding	< 4 weeks ago (*n* = 167)	28.7	34.1	31.7	5.4	< 0.001
	4+ weeks ago (*n* = 131)	57.3	24.4	16.0	2.3	
Child TV Viewing	< 4 weeks ago (*n* = 168)	55.4	28.0	15.5	1.2	< 0.001
	4+ weeks ago (*n* = 132)	78.0	12.9	9.1	0.0	
Child Physical Activity	< 4 weeks ago (*n* = 167)	36.5	35.3	23.4	4.8	< 0.001
	4+ weeks ago (*n* = 133)	70.7	15.0	13.5	0.8	

^a^Figures are percentages. †*P*-values are for comparison between groups based on duration since last attendance at mothers’ group

No differences in peer influence according to maternal age (< 35 years vs. ≥35 years), maternal BMI (healthy weight vs. overweight or obese) or child BMI Z-score (median split at z-BMI 0.67) were observed. (*p* > 0.05, data not displayed).

### Peer influences and behaviour

The only behaviours to be associated with peer influence using data from the total sample were a) breast feeding, where partner influence on attitudes was significantly associated (*p* = 0.004) and b) consumption of hot chips, where influence of other family on attitudes was significantly associated (*p* = 0.012). Mothers who rated their partners as either some influence on attitudes toward child feeding (average breastfeeding duration = 9.0 months) or a major influence (8.1 months) had significantly longer breastfeeding duration than mothers who rated partners as either a little influence (6.2 months) or no influence (6.7 months). No association was seen between the influence of any peer groups (mothers’ group, friends, partner, other family) on maternal attitudes, and other behaviours examined, including age of introduction of solids, amount of time child spends outside or the consumption of fruits or vegetables (all *p* > 0.05). When analyses were conducted using participants from the control group only (in order to exclude any potential impact of the InFANT intervention on results), the influence of partners on breastfeeding duration remained (*p* = 0.019), while the influence of other family on hot chip consumption was no longer significant (*p* = 0.31). Using the control group participants only, the only other behaviour to be influenced by peers was consumption of vegetables, with partner influence on attitudes being associated with consumption (*p* = 0.002).

## Discussion

Findings from this study suggest that maternal attitudes toward eating, physical activity and sedentary behaviours of infants are influenced by several external social influencers, with partners rated as the strongest influence. The influence of other new mothers from their mothers’ group was moderated by duration since last involvement with the group. In both the total sample, and when examining participants from the control group only, partners were the only peer group whose influence was found to be associated with measured behaviours, with greater influence on attitudes to child feeding associated with longer breastfeeding duration.

Other studies have had similar findings, with partner knowledge and support of breastfeeding being associated with higher breastfeeding initiation and continuation rates and several intervention trials targeting male partners having been successful in improving these outcomes [27]. While most literature, and our own findings, supports the proposition that supportive partners will improve breastfeeding outcomes, this is not uniformly consistent. For example, it has been suggested that practical support by fathers and grandmothers can be associated with lower rates of breastfeeding due to substitution of feeding responsibilities [14]. It has also been found that attitudes of grandmothers towards breastfeeding and complementary feeding can be inconsistent with the advice of health professionals and negatively influence a mothers’ decision to exclusively breastfeed [15]. Clearly, these findings suggest that family do have an impact on breastfeeding outcomes. While the direction of the association between partner support and maternal attitudes to breastfeeding could not be determined from this study, the association between partner influence and actual breastfeeding duration suggests that supportive male partners appear to have an overall positive impact.

While the role of fathers in complementary and later infant feeding has been less studied, current evidence suggests that fathers do play a role in shaping their child’s diet. Partner influence was significantly associated with control group children’s consumption of vegetables in our study, while a separate study of fathers involved in the Melbourne InFANT Program found a positive correlation between the healthiness of diets of fathers and their twenty-month-old children [10]. Mothers usually have greater responsibility and control over meal times than fathers but fathers are increasingly becoming more involved in child feeding [28]. It seems very likely that including fathers in nutrition-related obesity prevention interventions will be important to maximise intervention success.

We found no association between any peer influence and time spent outside by children in this study. Recent research by Schoeppe et al. showed that both parents’ own sedentary behaviours were positively associated with the sedentary behaviours of their children [29]. Another study on fathers’ perceptions toward child eating and physical activity behaviours suggested that fathers believe adequate physical activity is important for their young children and that they do play an important role in promoting these behaviours [30]. It is possible that our questions assessing influence of others on maternal attitudes does not correspond with the influence on actual behaviour, or that the direction of the influence of others was not universally positive.

After partners, self-reported influence on attitudes by other family members were reported as having the next greatest influence on child behaviours in this study. Mothers were not asked to specify which relatives they were referring to in this study, however current literature suggests that grandmothers often play the biggest role in the early stages of infant life [15] . The effect of a mother’s upbringing can also influence her own parenting behaviour [19] which may mean that this familial influence could also be part of what mothers meant by the influence of other family members in this study.

Education is a better measure of socioeconomic position in new mothers than income or occupation given the impact of child-rearing on these indices [31]. We found that more highly educated mothers were more likely to rate each of partners, mothers’ groups, other family and friends to be a significant influence on their child’s behaviours compared to less educated mothers. More highly educated mothers may either have stronger social networks overall, or draw on these networks more for parenting-related behaviours. Given the strong link between socioeconomic position and obesity-related behaviours [31], it is difficult to know whether the apparently weaker influence of social networks on attitudes of lower educated mothers are likely to be a net positive or negative influence on behaviours.

The finding that mothers in the intervention arm of the InFANT program were more likely to rate their mothers’ group as a significant influence on their child’s behaviours may reflect the intervention’s success in encouraging peer support and group discussion. It also suggests that the program is still having this effect two years post-intervention. More regular contact with mothers’ groups was associated with higher perceived influence of the group on feeding. We previously reported that breast feeding status of mothers’ group attendees (the true peer group) was a significant influence on how likely a mother was to continue to breast feed [16]. It was also demonstrated in that study that an infant’s peer interaction with other babies at age 4 months, and mother and baby physical activity levels at child age 9 months were positively associated with physical activity levels at 19 months [17]. High levels of participation and rates of continuation of mothers’ groups in an informal capacity suggests that mothers’ groups may be an important site for health promotion interventions [16].

### Strengths and limitations

A key strength of this research is that it includes child feeding, physical activity and sedentary behaviours. Limitations include the self-reported nature of some data collected, and the educated sample which may reduce the generalisability of the findings. Collection of data on the influence of others was years after collection of data on breastfeeding and introduction of solids due to the retrospective nature of the study design, and responses may have differed if these data were collected simultaneously. It is possible that recall/recency bias may have been a factor in mothers from active mothers’ groups rating the influence of these groups as stronger than mothers who were not actively meeting with mothers’ groups. No similar research has been undertaken previously, with further refinement of this tool potentially also including questions on the direction of the social influence – whether it was seen as being positive or negative. This could be particularly important given that even though here we found that associations between partner influence and breastfeeding duration were positive, separate qualitative research among 26 mothers from the InFANT Program has found the influence of partners on later feeding practices was frequently negative [19]. How to identify mothers who are at risk due to either a lack of positive social influencers or the presence of negative influencers could be an important topic for further research.

## Conclusions

Infant health behaviours are influenced by maternal peers, with our study finding that mothers report partners as being the most influential on their attitudes toward their child’s feeding, physical activity and sedentary behaviours. This adds to the current evidence that partner behaviour is an important determinant of infant health and partners could play an important role in obesity prevention interventions. Our results showed that the influence of peers and family members on attitudes to obesity-related behaviours is stronger for mothers with higher levels of education. Research into why peer influences might be socioeconomically patterned, and the consequences of this for child behaviour is required. Although we found that the level of partner influence on child feeding was associated with breastfeeding duration, further research on whether different peers are a positive or negative influence on child behaviours would also be valuable and would help to more effectively target obesity prevention interventions.

## Supplementary information

**Additional file 1.** Contains a copy of the questionnaires used in this study to assess the influence of the maternal peer group (partner, friends, mothers’ group, family) on mothers’ attitudes to obesity-related behaviours of their children.

## Data Availability

The datasets supporting the conclusions of this article and/or analyzed during the current study are available from the corresponding author on reasonable request.

## Abbreviations

BMI: Body Mass Index
WHO: World Health Organization

## References

[1] Australian Institute of Health and Welfare. Overweight and Obesity. 2019. Available from: http://www.aihw.gov.au/overweight-and-obesity/. Accessed 7 Oct 2019.

[2] Gibbs BG, Forste R (2014). Socioeconomic status, infant feeding practices and early childhood obesity†. Pediatric Obesity.

[3] Camargos ACR, Mendonça VA, Andrade CA, Oliveira KSC, Lacerda ACR (2016). Overweight and obese infants present lower cognitive and motor development scores than normal-weight peers. Res Dev Disabil.

[4] Sanders RH, Han A, Baker JS, Cobley S (2015). Childhood obesity and its physical and psychological co-morbidities: a systematic review of Australian children and adolescents. Eur J Pediatr.

[5] Boak R, Virgo-Milton M, Hoare A, Silva A, Gibbs L, Gold L, Gussy M, Calache H, Smith M, Waters E (2016). Choosing foods for infants: a qualitative study of the factors that influence mothers. Child Care Health Dev.

[6] Spence AC, Campbell KJ, Crawford DA, McNaughton SA, Hesketh KD (2014). Mediators of improved child diet quality following a health promotion intervention: the Melbourne InFANT program. Int J Behav Nutr Phys Activ.

[7] Fulkerson JA, Larson N, Horning M, Neumark-Sztainer D (2014). A review of associations between family or shared meal frequency and dietary and weight status outcomes across the lifespan. J Nutr Educ Behav.

[8] Huilan X, Li Ming W, Hardy LL, Rissel C (2016). A 5-year longitudinal analysis of modifiable predictors for outdoor play and screen-time of 2- to 5-year-olds. Int J Behav Nutr Phys Activ.

[9] Xu H, Wen LM, Rissel C (2014). Associations of maternal influences with outdoor play and screen time of two-year-olds: findings from the healthy beginnings trial. J Paediatr Child Health.

[10] Walsh AD, Cameron AJ, Hesketh KD, Crawford D, Campbell KJ (2015). Associations between dietary intakes of first-time fathers and their 20-month-old children are moderated by fathers’ BMI, education and age. Br J Nutr.

[11] Vollmer RL, Adamsons K, Gorin A, Foster JS, Mobley AR (2015). Investigating the Relationship of Body Mass Index, Diet Quality, and Physical Activity Level between Fathers and Their Preschool-Aged Children. J Acad Nutr Diet.

[12] Vollmer RL, Adamsons K, Foster JS, Mobley AR (2015). Association of fathers' feeding practices and feeding style on preschool age children's diet quality, eating behavior and body mass index. Appetite.

[13] Bilal SM, Dinant G, Blanco R, Crutzen R, Mulugeta A, Spigt M (2016). The influence of father's child feeding knowledge and practices on children's dietary diversity: a study in urban and rural districts of northern Ethiopia, 2013. Matern Child Nutr.

[14] Emmott EH, Mace R (2015). Practical support from fathers and grandmothers is associated with lower levels of breastfeeding in the UK millennium cohort study. PLoS One.

[15] Kuswara K, Laws R, Kremer P, Hesketh KD, Campbell KJ (2016). The infant feeding practices of Chinese immigrant mothers in Australia: a qualitative exploration. Appetite.

[16] Cameron AJ, Hesketh K, Ball K, Crawford D, Campbell KJ (2010). Influence of peers on breastfeeding discontinuation among new parents: the Melbourne InFANT program. Pediatrics.

[17] Hnatiuk J, Salmon J, Campbell KJ, Ridgers ND, Hesketh KD (2013). Early childhood predictors of toddlers’ physical activity: longitudinal findings from the Melbourne InFANT program. Int J Behav Nutr Phys Act.

[18] Bai L, Fong Y, Lok Y, Tarrant M (2016). Relationship between the infant feeding preferences of Chinese mothers' immediate social network and early breastfeeding cessation. J Hum Lact.

[19] Spence AC, Hesketh KD, Crawford DA, Campbell KJ (2016). Mothers’ perceptions of the influences on their child feeding practices – a qualitative study. Appetite.

[20] Christakis NA, Fowler JH (2007). The spread of obesity in a large social network over 32 years. N Engl J Med.

[21] Trogdon JG, Allaire BT (2014). The effect of friend selection on social influences in obesity. Econ Hum Biol.

[22] Pachucki MA, Jacques PF, Christakis NA (2011). Social network concordance in food choice among spouses, friends, and siblings. Am J Public Health.

[23] Bahr DB, Browning RC, Wyatt HR, Hill JO (2009). Exploiting social networks to mitigate the obesity epidemic. Obesity.

[24] Hesketh KD, Campbell K, Salmon J, McNaughton SA, McCallum Z, Cameron A, Ball K, Gold L, Andrianopoulos N, Crawford D (2013). The Melbourne infant feeding, activity and nutrition trial (InFANT) program follow-up. Contemp Clin Trials.

[25] Campbell K, Hesketh K, Crawford D, Salmon J, Ball K, McCallum Z (2008). The infant feeding activity and nutrition trial (INFANT) an early intervention to prevent childhood obesity: cluster-randomised controlled trial. BMC Public Health.

[26] WHO child growth standards: Length/height-for-age, weight-for-age, weight-for-length, weight-for-height and body mass index-for-age: Methods and development. Accessed 24/5/19. [http://www.who.int/childgrowth/standards/technical_report/en/].

[27] Mitchell-Box KM, Braun KL (2013). Impact of male-partner-focused interventions on breastfeeding initiation, exclusivity, and continuation. J Hum Lact.

[28] Mallan KM, Nothard M, Thorpe K, Nicholson JM, Wilson A, Scuffham PA, Daniels LA (2014). The role of fathers in child feeding: perceived responsibility and predictors of participation. Child Care Health Dev.

[29] Schoeppe S, Vandelanotte C, Bere E, Lien N, Verloigne M, Kovács É, Manios Y, Bjelland M, Vik FN, Van Lippevelde W. The influence of parental modelling on children's physical activity and screen time: does it differ by gender? Eur J Public Health. 2017;27(1):152–7. 10.1093/eurpub/ckw182 28177458

[30] Walsh AD, Hesketh KD, van der Pligt P, Cameron AJ, Crawford D, Campbell KJ (2017). Fathers’ perspectives on the diets and physical activity behaviours of their young children. PLoS One.

[31] Cameron AJ, Spence AC, Laws R, Hesketh KD, Lioret S, Campbell KJ (2015). A review of the relationship between socioeconomic position and the early-life predictors of obesity. Curr Obes Rep.

