# Supplementary material for: The influence of the maternal peer group (partner, friends, mothers’ group, family) on mothers’ attitudes to obesity-related behaviours of their children
Source: BMC Pediatr. 2019 Oct 16;19:357. doi: 10.1186/s12887-019-1726-x (PMC6794892; doi:10.1186/s12887-019-1726-x)
Supplement: Supplementary file 1 — Additional file 1. Contains a copy of the questionnaires used in this study to assess the influence of the maternal peer group (partner, friends, mothers’ group, family) on mothers’ attitudes to obesity-related behaviours of their children. [file 12887_2019_1726_MOESM1_ESM.docx]

**Supplementary file** – questionnaire used in this study to assess the influence of the maternal peer group (partner, friends, mothers’ group, family) on mothers’ attitudes to obesity-related behaviours of their children

**QE9     How long has it been since you met regularly with any members of your mothers group?**

            _____weeks          **OR**      _____months

**QE10   How much of an influence on your attitudes towards *feeding* your child have the following been?** (Please shade *one* response on each line)

|  | **No Influence** | **A Little Influence** | **Some Influence** | **Major Influence** |  |
| --- | --- | --- | --- | --- | --- |
| **a.**  Mothers group | ⭘ _1_ | ⭘ _2_ | ⭘ _3_ | ⭘ _4_ |  |
| **b.**  Friends | ⭘ _1_ | ⭘ _2_ | ⭘ _3_ | ⭘ _4_ |  |
| **c.**  Partner | ⭘ _1_ | ⭘ _2_ | ⭘ _3_ | ⭘ _4_ | I don’t have a partner ⭘ _5_ |
| **d.** Other family members | ⭘ _1_ | ⭘ _2_ | ⭘ _3_ | ⭘ _4_ |  |

**QE11   How much of an influence on your attitudes towards your child’s *television viewing* have the following been?** (Please shade *one* response on each line)

|  | **No Influence** | **A Little Influence** | **Some Influence** | **Major Influence** |  |
| --- | --- | --- | --- | --- | --- |
| **a.**  Mothers group | ⭘ _1_ | ⭘ _2_ | ⭘ _3_ | ⭘ _4_ |  |
| **b.**  Friends | ⭘ _1_ | ⭘ _2_ | ⭘ _3_ | ⭘ _4_ |  |
| **c.**  Partner | ⭘ _1_ | ⭘ _2_ | ⭘ _3_ | ⭘ _4_ | I don’t have a partner ⭘ _5_ |
| **d.** Other family members | ⭘ _1_ | ⭘ _2_ | ⭘ _3_ | ⭘ _4_ |  |

**QE12   How much of an influence on your attitudes towards your child’s *activity levels* have the following been?** (Please shade *one* response on each line)

|  | **No Influence** | **A Little Influence** | **Some Influence** | **Major Influence** |  |
| --- | --- | --- | --- | --- | --- |
| **a.**  Mothers group | ⭘ _1_ | ⭘ _2_ | ⭘ _3_ | ⭘ _4_ |  |
| **b.**  Friends | ⭘ _1_ | ⭘ _2_ | ⭘ _3_ | ⭘ _4_ |  |
| **c.**  Partner | ⭘ _1_ | ⭘ _2_ | ⭘ _3_ | ⭘ _4_ | I don’t have a partner ⭘ _5_ |
| **d.** Other family members | ⭘ _1_ | ⭘ _2_ | ⭘ _3_ | ⭘ _4_ |  |
